# Integral Roles for Integrins in γδ T Cell Function

**DOI:** 10.3389/fimmu.2018.00521

**Published:** 2018-03-13

**Authors:** Gabrielle M. Siegers

**Affiliations:** ^1^Experimental Oncology, University of Alberta, Edmonton, AB, Canada

**Keywords:** gamma delta T cells, adhesion and signaling molecules, cellular migration, tissue retention, tissue localization, tumor infiltrating lymphocytes, cytotoxicity, cytokine secretion

## Abstract

Integrins are adhesion receptors on the cell surface that enable cells to respond to their environment. Most integrins are heterodimers, comprising α and β type I transmembrane glycoprotein chains with large extracellular domains and short cytoplasmic tails. Integrins deliver signals through multiprotein complexes at the cell surface, which interact with cytoskeletal and signaling proteins to influence gene expression, cell proliferation, morphology, and migration. Integrin expression on γδ T cells (γδTc) has not been systematically investigated; however, reports in the literature dating back to the early 1990s reveal an understated role for integrins in γδTc function. Over the years, integrins have been investigated on resting and/or activated peripheral blood-derived polyclonal γδTc, γδTc clones, as well as γδ T intraepithelial lymphocytes. Differences in integrin expression have been found between αβ T cells (αβTc) and γδTc, as well as between Vδ1 and Vδ2 γδTc. While most studies have focused on human γδTc, research has also been carried out in mouse and bovine models. Roles attributed to γδTc integrins include adhesion, signaling, activation, migration, tissue localization, tissue retention, cell spreading, cytokine secretion, tumor infiltration, and involvement in tumor cell killing. This review attempts to encompass all reports of integrins expressed on γδTc published prior to December 2017, highlights areas warranting further investigation, and discusses the relevance of integrin expression for γδTc function.

## Introduction

Although much was known about integrins on lymphocytes as early as 1990 (1), integrin expression on γδTc has been only sporadically, and often indirectly, investigated. Considered all together, these reports reveal an understated role for integrins in γδTc function (Table 1).

**Table 1:** Integrin expression reported on γδ T cells; cells used were blood-derived unless otherwise indicated.

	α	β	a.k.a	Binds	Function	spp	Reference
**β1**							
α1β1	CD49a	CD29	VLA-1	Collagen IV	Extravasation, tumor infiltration, cellular morphology	H	(16)
α2β1	CD49b	CD29	VLA-2	Collagen	n.d.	H	(15)
α4β1	CD49d	CD29	VLA-4	FN	n.d.	H	(15)
					Signaling, adhesion	H	(17)
					Adhesion to endothelial cells	H	(9)
				VCAM-1	Endothelial layer permeability	H	(29)
					Transendothelial migration?	H	(30)
					Adhesion to fibroblasts	H	(49)

α5β1	CD49e	CD29	VLA-5	FN	n.d.	H	(15)
					Signaling, adhesion	H	(17)
					Transendothelial migration?	H	(30)
					Vδ1 activation, localization, retention	H	(9)
					Adhesion to fibroblasts	H	(49)

α6β1	CD49f	CD29	VLA-6		Transendothelial migration	H	(30)

**β2**							
αLβ2	CD11a	CD18	LFA-1	CD54/ICAM-1	Adhesion to endothelial and epithelial cells, fibroblasts	H	(9)
					Naive αβTc activation?	H	(19)
					Endothelial layer permeability	H	(29)
					Transendothelial migration?	H	(30)
					Trafficking to infected airways (TB)?	NHP	(33)
					Adhesion to fibroblasts	H	(49)
					K562 leukemia cell binding	H	(54)
					Cytotoxicity against Burkitt Lymphoma, prostate cancer, Daudi B cell lymphoma	H	(55–58)
					CNS trafficking in EAE? (LN, spleen-derived)	M	(22)

αMβ2	CD11b	CD18	Mac-1		Naive αβTc activation?	H	(19)
			Mo-1		Early fetal thymocyte differentiation?	M	(67)
					CNS trafficking in EAE? (LN, spleen-derived)	M	(22)

αXβ2	CD11c	CD18	P150,95		Naive αβTc activation?	H	(19)
					Homing, activation, interferon γ secretion	H	(20)
					CNS trafficking in EAE? (LN, spleen-derived)	M	(22)

αDβ2	CD11d	CD18		ICAM-1	Vδ1 cell spreading?	H	(25)
				VCAM-1	Inflammatory response? Vδ1 tissue retention?	H	(23)
					Proliferation?	M	(22)
					Early fetal thymocyte differentiation?	M	(67)
					CNS trafficking in EAE? (LN, spleen-derived)	M	(22)

**β3**							
αvβ3	αv	β3	VNR	RGD sequence	IL-4 production (DETC)	M	(71)

**β7**							
αEβ7	CD103	β7		E-cadherin	Epithelial retention of γδTc IEL?	H	(37)
					M	(78, 79)
					Proliferation? IL-9 production?	H	(40)
					Vδ1 binding SCC	H	(49)
					Vδ1 tumor retention?	H	(49)
					Homing to gut? (mLN, colitis)	M	(80)
					Homing to and retention in gut?	R	(81)

α4β7	CD49d	β7		MadCAM	Susceptibility to HIV infection on CCR5^+^Vδ2	H	(60)
					Homing to gut (TDL, RTE)	M	(76, 80)
					Migration to inflamed tissue in allergic reaction	M	(77)
					Migration to tissues	B	(7)

*Question marks denote suggested functions that require further validation. a.k.a., also known as; B, bovine; CNS, central nervous system; DETC, dendritic epidermal T cells; EAE, experimental autoimmune encephalitis; ECM, extracellular matrix; FN, fibronectin; H, human; ICAM, intercellular adhesion molecule; IEL, intraepithelial lymphocyte; IL, interleukin; LFA-1, lymphocyte function-associated antigen-1; LN, lymph node; M, murine; MAdCAM-1, mucosal addressin cell adhesion molecule 1; mLN, mesenteric lymph node; n.d., not determined in this report (with respect to γδ T cells); NHP, nonhuman primate; ref, reference; RTE, recent thymic emigrant; SCC, squamous cell carcinoma; spp, species; TB, tuberculosis; TDL, thoracic duct lymphocytes; VCAM-1, vascular cell adhesion molecule-1; VLA, very late antigen; VNR, vitronectin receptor*.

Integrins are heterodimeric adhesion receptors comprising non-covalently linked α and β chains (2). Greek letters indicating chain pairings for β1 and cluster of differentiation designations for β2 integrins are used throughout this review; cited works may have used alternative nomenclature.

## Integrin Activation and Functions

Integrins play a role in many cellular functions including development, activation, differentiation, proliferation, mobility, and survival (1, 3). Integrins enable two-way communication between cells (cytoskeleton) and their surroundings [extracellular matrix (ECM), other cells]. ECM proteins with which integrins interact include collagen, a structural protein, and adhesion proteins fibronectin (FN) and vitronectin (4). Signaling through integrins can be “inside-out,” regulating extracellular interaction between integrins and their ligands, but also “outside-in,” influencing actin cytoskeleton rearrangement as well as gene expression and transcription of associated proteins, including cytokines, to impact cellular processes (5, 6).

Integrins are integral to lymphocyte homing to tissues and migration within tissues; they—together with selectins and their respective ligands—participate in tethering, rolling, and adhesion (7). Integrins respond to chemokine signaling arresting migration of lymphocytes and facilitating transmigration into tissues (8). In contrast to other cell types, β1 integrins on conventional T cells require activation for adhesion to occur (9, 10). Basal adhesion levels reflect inactive or low-affinity status of integrins; stimulus with 12-O-tetradecanoylphorbol-13-acetate, anti-CD3 or anti-CD2 activates β1 integrins, converting them to a high-affinity state without necessitating greater surface expression (10). Such activation dependence is also true for the β2 integrin CD11a/CD18 in T cell adhesion and de-adhesion (11). Indeed, several integrins serve as costimulatory molecules in concert with T cell antigen receptor (TCR) engagement (10, 12–14). Much occurs downstream of integrin-mediated cell adhesion, including phosphorylation of proteins in signaling pathways for cell cycle, cytokine expression, and cytoskeletal remodeling enabling processes such as proliferation and migration (3, 6).

**Figure 1:**
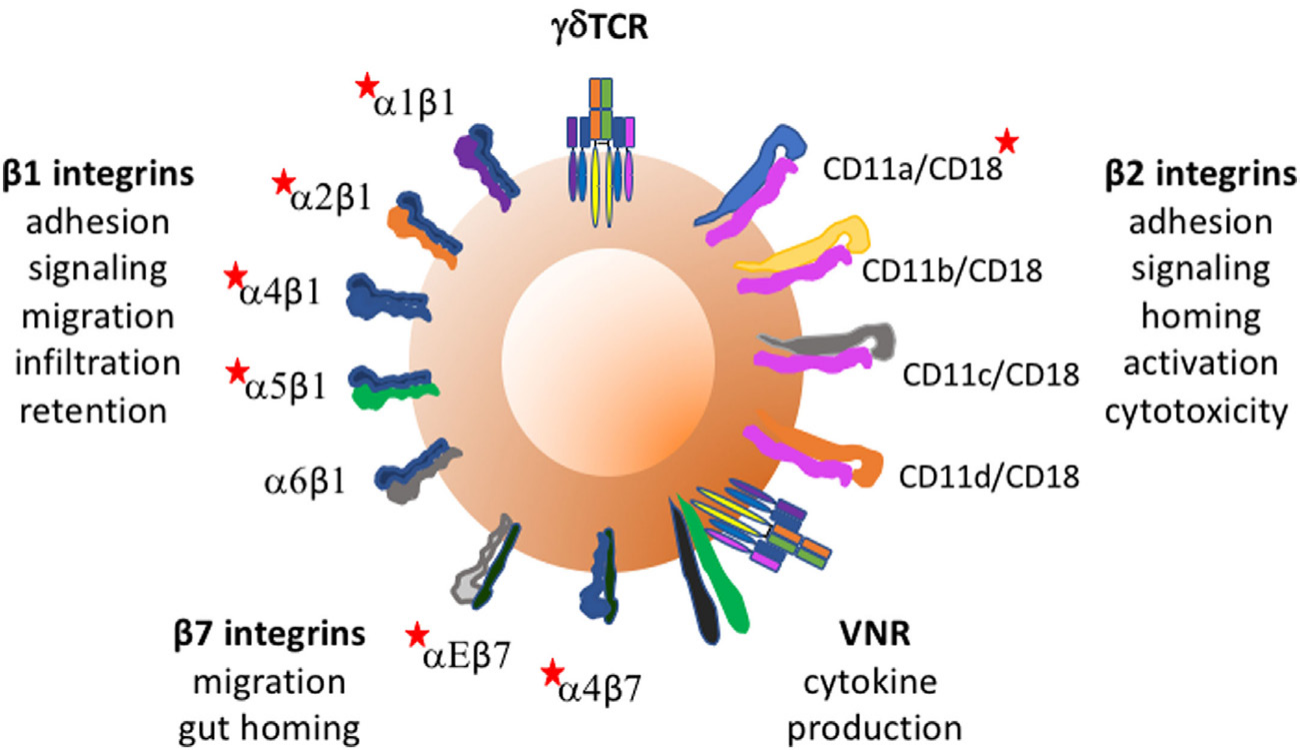
Integrins found on γδ T cells and some of their functions. Red stars indicate integrins whose expression and/or function on γδTc has been reported to require activation. Vitronectin receptor signals through CD3 zeta of the TCR.

Integrins on human γδTc will first be considered, loosely grouped according to function, and then findings in other species will be discussed. Figure 1 depicts integrins found on γδTc and some of their functions.

## Adhesion and Signaling

In 1992, α4, β1, and CD18 were identified on human Vγ9 γδTc derived from stimulated peripheral blood mononuclear cells (PBMCs). While no α3, αv, or β3 expression was observed, less than 30% expressed α1, α2, or α5 chains. CD8^+^ γδTc clones expressed high β1, and consistent α4 and α5 levels. Phorbol 12-myristate 13-acetate (PMA)-induced adhesion *via* integrin activation; while α2β1 was required for collagen binding, FN binding relied on both α4β1 and α5β1. Most polyclonal γδTc only expressed α4β1, whereas individual clones showed variation attributed to extended culturing and selection during cloning (15), corroborating evidence that β1 expression on T cells increases qualitatively and quantitatively over time in culture (1, 16). Admittedly, these studies used activated γδTc and may not have reflected the state of cells in circulation (15).

Expression of α4 and α5 on CD3^+^CD4^−^CD8^−^ γδTc, and lack of α3 or α6 was confirmed. Activated CD25^hi^ γδTc bound FN better than resting CD25^low^ γδTc, mediated mostly by α4 and partly by α5. Culturing cells on immobilized anti-γδ TCR antibodies together with FN enhanced proliferation and increased CD25 expression, suggesting both signaling and adhesion roles for α4 and α5 integrins. While γδTc adhesion required activation through the TCR, surface levels of α4 and α5 remained unaltered (17). Cytokines such as interleukin (IL)-1β and TNF-α may influence γδTc integrin expression and/or activation (18); this has yet to be explored.

Compared to αβTc, fresh primary γδTc were more adhesive (~2:1 to 4:1) to endothelial cells, fibroblasts, and epithelial cells independent of activation. Both αβTc and γδTc required CD11a/CD18 and α4β1 to bind endothelial cells, whereas CD11a/CD18-ICAM-1 interaction facilitated adherence to fibroblasts and epithelial cells. Phorbol dibutyrate treatment of PBMCs and cytokine stimulation of monolayers greatly enhanced T cell adhesion, correlated with their expression of CD11a/CD18 and α4β1 (9). CD11a, b, c, and CD18 were detected on isopentenyl pyrophosphate (IPP)-stimulated γδTc, in parallel with markers indicating antigen presenting potential; integrins were likely involved in clustering between γδTc and naïve αβTc in an activation capacity, but their role was not directly addressed (19). It would be of interest to determine whether loss of one or more integrins might impact γδTc antigen presentation.

In healthy women, constitutively high CD11c levels were observed on circulating CCR7^−^CD4^−^ populations co-expressing γδTCR and CD8; cervical γδTc (>20%) also expressed CD11c. α1β1 and α4β7 were co-expressed on CD11c^+^CCR7^−^CD4^−^ T cells, of which γδTc were a part, but unfortunately not specifically analyzed. CD11c expression was associated with T cell homing and activation, and interferon γ (IFNγ) secretion in a fraction of (E)-4-hydroxy-3-methyl-but-2-enyl pyrophosphate-stimulated γδTc (20).

CD11d, first described in 1995 (21), has now been identified on both murine (22) and human γδTc (23). CD11d/CD18 binds vascular cell adhesion molecule (VCAM-)1 (24) and intercellular adhesion molecule (ICAM-)3 (21). Vδ1 clones cultured on anti-ICAM-3 plates in the presence of IL-2 underwent spreading; however, the participating receptor on γδTc had not yet been identified (25). Since ICAM-3 is a CD11d ligand, and CD11d is highly expressed on Vδ1 γδTc (23), it was likely CD11d-ICAM-3 interaction mediating this response. ICAM-3 may play a role in inflammatory response initiation, potentially aiding in such processes as antigen presentation and cytotoxicity (26). ICAM-3 on neutrophils participates in IFNγ production but not cytotoxicity of NK cells (27) and has some predictive value in perioperative systemic inflammatory response syndrome (28). Thus, CD11d on γδTc may play a role in inflammation, but this requires further investigation.

## Transendothelial Migration

In the first report investigating mechanisms by which γδTc cross the endothelium to migrate into inflamed tissue from the circulation, CD11a/CD18 and α4β1 on γδTc bound to endothelial cell ligands CD54/ICAM-1 and VCAM-1, respectively, increasing endothelial cell permeability. While cytotoxicity of γδTc clones to endothelial cells surely contributed to endothelial layer permeability, it was thought unlikely to occur with autologous cells *in vivo* (29).

An immunophenotyping study showed that γδTc had greater transendothelial migratory capacity than αβTc (30), explaining γδTc enrichment in chronic inflammation (31, 32), attributed to CD11a/CD18 expression, and increased α4, α5, and α6 β1 integrin density on migrating compared to non-migrating T cells; blocking assays were not performed to confirm functional relevance here (30). While CD11d expression on PBMC-derived γδTc was higher compared to αβTc (freshly isolated or expanded), their migratory ability was not compared (23). In a non-human primate tuberculosis model, adoptively transferred Vδ2 cells trafficking to infected airways expressed CD11a/CD18 (33). In contrast, increased numbers of peripheral γδTc expressing reduced CD18 levels were identified in patients suffering acute psoriasis, suggesting a role in disease pathogenesis (34).

## Integrins on Vδ1 Versus Vδ2 Directing Localization and Tissue Retention

Integrins likely play a role in the tissue specificity of γδTc subsets. In Galéa’s study, Vδ1 and Vδ2 migrated similarly, suggesting that Vδ1 tissue accumulation relates to their retention rather than migratory abilities (30). Indeed, higher CD11d expression on Vδ1 compared to Vδ2 cells may also account for preferential Vδ1 retention (23), as well as Vδ1 prevalence in large intestinal mucosal epithelium (35) and conditions such as rheumatoid arthritis (31, 32, 36).

An E-cadherin binding integrin associated with epithelial retention, αEβ7 (CD103), was found on human γδTc intraepithelial lymphocytes (IELs). While peripheral blood T cells did not express much αEβ7 the authors posited its upregulation after T cells extravasate in the lamina propria, since αEβ7 expression positively correlated with nearer proximity to epithelium (37). IL-2 and phytohemagglutinin (PHA) stimulation activated αEβ7 on cultured CD4^+^CD8^+^ IEL, and TCR crosslinking enhanced αEβ7-E-cadherin avidity (38). On αβTc, this transforming growth factor β (TGF-β)-induced integrin is associated with pro- and anti-inflammatory conditions, tissue retention, and both cytotoxic and regulatory T lymphocyte tumor infiltration and function, expertly reviewed in Ref. (39). Peters and colleagues noted upregulation of *ITGAE*, the gene encoding αEβ7, and corresponding surface expression on expanded Vδ2 cells treated with TGF-β and IL-15 correlating with enhanced proliferation and IL-9 production (40).

Subset variation exists for α5β1, with Vδ1 expressing more than Vδ2, providing an explanation for previous reports of low α5β1 expression in studies focusing on Vδ2 cells. High α5β1 expression accounted for increased Vδ1 binding to FN, potentially reflecting Vδ1 adhesion to fibroblasts *in vivo*, and the importance of this interaction for Vδ1 activation and localization (9). During inflammation, mucosal epithelial cells display increased FN levels (41), which may increase Vδ1 retention. Similar ICAM-1 and VCAM-1-mediated binding of Vδ1 and Vδ2 cells could be explained by their comparable expression of CD11a/CD18 and α4β1 (9). Thus, γδTc tissue recruitment may be achieved through CD11a/CD18 and α4β1 binding to endothelial cell ligands, and cells retained in tissue *via* CD11a/CD18 and α5β1 interactions with epithelial cell-, fibroblast-, and ECM ligands (9).

## Tumor Infiltration

Increased α1β1 expression may facilitate γδTc migration out of vessels and infiltration into tumors (16). A known receptor for the basement membrane protein collagen IV, α1β1 has been observed on IL-2-activated T cells invading tumors (42–47). While resting peripheral blood T cells expressed little α1β1, its expression increased over time in culture; γδTc clones expressed higher α1β1 than polyclonal T cells (16), corroborating observed α1β1 expression on long-term activated T cells (48). Anti-α1β1 inhibited CD8^+^ γδTc interaction with collagen IV, but not FN or collagen I, in a concentration-dependent manner. Cellular morphology was impacted, as Mg^2+^ cation-dependent spreading of long-term cultured CD8^+^ α1β1^high^ αβTc or γδTc on collagen IV-coated slides was inhibited by anti-α1β1 antibodies (16).

Compared to αβTc, γδTc derived from patient blood bound squamous carcinoma (SCC) and fibroblast cells more tightly (49), confirming previous results (9). While CD11a/CD18 played a role in both cases, SCC binding was mediated *via* l-selectin and CD44v6; fibroblast binding was achieved though α4β1 and α5β1 (49).

Vδ1 predominance has been reported in tumor infiltrating lymphocytes from lung (50), colon (51), renal carcinoma (52), and esophageal tumors (49). Preferential extravasation, infiltration, and retention of Vδ1 cells in esophageal tumors was attributed to higher expression and a greater variety of integrins such as CD11a/CD18, α4β1, α5β1, and αΕβ7 on Vδ1 compared to Vδ2. In particular, Vδ1 used αΕβ7 to bind SCC. Since esophageal tumors also express E-cadherin, αΕβ7 expression may provide a mechanism of lymphocyte retention in tumors (49).

## Cytotoxicity

CD11a/CD18 facilitates effector-target cell conjugation (53). This interaction, integral to γδTc cytotoxicity, has been confirmed in binding assays with K562 leukemia (54), and blocking assays with Burkitt Lymphoma (55), prostate cancer (56, 57), and Daudi B cell lymphoma cells (58). We have observed significant γδTc apoptosis induced by anti-γδTCR (59) antibodies; thus, this may also occur with antibodies blocking CD18 and should be considered when designing controls and interpreting results from blocking assays using such antibodies. Activation of αβTCR changes CD11a/CD18 avidity from low to high transiently, to allow adhesion, but then also de-adhesion of T cells, promoting continued serial killing (11). If this holds true for the γδTCR, then this mechanism greatly contributes to γδTc cytotoxicity and could be therapeutically relevant.

## Susceptibility to Viral Infection

In the absence of CD4, high α4 and β7 levels on IPP-expanded Vδ2 cells formed a complex with high levels of CCR5 (fivefold higher than αβTc); this inferred HIV envelope glycoprotein susceptibility resulting in CD4^−^ Vδ2 cells’ demise (60). While Vδ1 express as much α4β7 as Vδ2, they do not express CCR5, thus rendering Vδ1 immune to HIV-envelope-mediated killing (61).

## Immunological Memory

CD11b (complement receptor 3, Mac-1) expression on peripheral blood T cells increased with age, leveling out later in life. γδTc expressed more CD11b than αβTc across all ages; and while not shown, CD11b was thought important for migration to spleen and liver, and to indicate antigen-specific memory T cells (62). Later studies suggested roles associated with T cell immunoregulation, proliferation, and homing (63, 64), but the significance of CD11b on human γδTc remains unknown. Increased αβTc integrin levels and adherence have been associated with memory CD4^+^ T cells (10, 65), but the only study addressing this with respect to γδTc equated Vδ1 with naïve and Vδ2 with memory cells, then compared Vδ1 to Vδ2 expression of CD11a, α4β1, and α5β1 (not CD11b), finding no correlation between adhesion/integrin levels and maturation (9). A longitudinal study following integrin expression and function during the course of γδTc maturation would be more appropriate to address this question, keeping in mind the influence of *in vitro* culture.

## Of Rodents and Ruminants in Health and Disease…

### β1 Integrins

In mice, β1 integrins play an important role in thymocyte differentiation into CD4^+^ and CD8^+^ αβTc; however, their role in γδTc development remains unknown (66).

### β2 Integrins

While not found on thymocytes in adult wild-type mice, transient co-expression of CD11b and CD11d on fetal thymocytes suggests an important role for β2 integrins in early differentiation (67).

In the context of experimental autoimmune encephalitis (EAE), murine γδTc differentially expressed β2 integrins and produced more IFNγ and tumor necrosis factor α in lymph nodes, spleen, and spinal cord compared to αβTc (22). At baseline, most γδTc expressed CD11a, b, and d. Both γδTc frequency and upregulation of β2 integrins, including CD11c, were noted after EAE induction; γδTc infiltration of the central nervous system (CNS) followed that of αβTc, but was more rapid (22). Thus, β2 integrin expression on γδTc affected their trafficking into the CNS, thereby impacting EAE development kinetics (22). In a follow-up study, EAE disease severity was similar in γδTc^−/−^ mice reconstituted with γδTc lacking CD11a, b, or c suggesting that β2 integrins were not important for CNS trafficking; however, CD11d was still present on γδTc, pointing to this integrin’s potential role in trafficking. CD11a/CD18^−/−^ γδTc displayed reduced CNS retention and expansion during EAE, suggesting CD11a involvement in both retention and co-stimulation (68). While not specific to γδTc, it is interesting that CD3 expression was reduced in CD11b^−/−^ and CD11d^−/−^ mice compared to wildtype. Furthermore, CD11b and CD11d seem important for proliferation of murine T cells stimulated with PHA and Concanavalin A or superantigen, but not for their response to PMA (67). Indeed, β2 integrin expression seems concomitant with T cell expansion, in line with observations of increased CD11d expression on human γδTc expanded under higher IL-2 levels (23). In a murine spontaneous psoriasis model, reduced CD18 resulted in loss of Vγ5^+^ skin-resident γδTc and expansion of lymph node derived skin-homing Vγ4^+^ γδTc contributing to disease initiation and progression. CD18^low^ γδTc expressed higher IL-7Rα levels and increased IL-7-induced proliferation generating inflammatory memory CD44^+^CD27^−^ capable of IL-17 production (34). Adoptive transfer experiments confirmed that low levels of CD18 did not impair γδTc trafficking to the skin in healthy mice (34). *Itgax*, the gene encoding integrin CD11c, is common to γδTc and NK cells, yet, differentiates γδTc from iNKT and αβTc in the mouse (69). Murine CD11c was identified on γδTc in the blood and genital tract, most predominantly on γδTc co-expressing NK1.1. Vaginal *Chlamydia* infection expanded circulating CD11c^+^ γδTc (20).

### The Vitronectin Receptor (VNR)

An integrin later identified as the VNR, or αvβ3, was found on murine dendritic epidermal T cell lines (DETC); its expression on splenic T cells was only observed after a minimum of 1 week of stimulation (70). VNR expression was soon further confirmed on autoreactive DETC-derived cell lines (6, 71, 72). A subset of these γδTc (Vγ1.1/Cγ4-Vδ6/Cδ1) secreted IL-4 in a VNR-dependent manner (71). In a follow-up report using a TCR^−/−^ hybridoma line transfected with CD3ζ fusion proteins, VNR- but not TCR-engagement by ligand was required in conjunction with CD3ζ chain signaling for IL-2 production (73). VNR recognizes the adhesive peptide sequence RGD in ECM proteins (74). While human αβTc can be induced to express VNR upon stimulation with PHA and/or PMA (75), VNR has not been found on polyclonal or clonal human γδTc (15).

### α4β7 and αEβ7

High levels of α4β7 were associated with gut-tropism of murine γδTc trafficking from adult thymus to the small intestine epithelium, whereupon reaching their destination, α4β7 was subsequently downregulated, through interaction with its counterreceptor mucosal addressin cell adhesion molecule 1 (MadCAM) on the lamina propria (76). In a model of allergic reaction, IL-17^+^ γδTc expressed α4β7 that enabled their mobilization by CCL25 in inflamed tissue, which in turn modulated IL-17 levels (77). Blocking α4β7 *in vivo* prevented the migration of IL-17^+^ γδTc but not αβTc into mouse pleura, and also blocked transmigration of γδTc across VCAM-1- and MadCAM-1-expressing endothelium toward CCL25 or cell-free pleural washes from mice in whom an allergic reaction had been induced (77). Bovine peripheral blood-derived CD8^+^ γδTc accumulated in MAdCAM-1-expressing tissues in a dose-dependent manner. CD8^+^ γδTc expressed 1.5-fold more α4β7 than CD8^–^ γδTc but similar β_1_ and β_2_ levels. While adding CXCL12 increased MAdCAM binding of all γδTc, CCL21 activated integrins and increased CD8^+^ γδTc binding to recombinant MAdCAM1 more so than CD8^−^ γδTc. Circulating human CD8^−^ and CD8^+^ γδTc migrated similarly in response to CCL21, and expressed comparable α4β7; this species-specific discrepancy was attributed to CD8 chain usage differences in humans (αα) versus cows (αβ) (7).

Prevalence of “inflammatory” γδTc (iγδTc) co-expressing high levels of gut-homing α4β7 and αEβ7 correlated with disease severity in both spontaneous and induced murine colitis models. Cytotoxicity, cytokine production, and NK cell receptor genes were upregulated on iγδTc compared to other γδTc subsets (expressing α4β7 or αEβ7) isolated from mesenteric lymph nodes in induced colitis, suggesting profound functional relevance of integrin co-expression on these cells (78).

In αEβ7-knockout mice, γδTc IEL migration within the intraepithelial compartment was enhanced (79) and remained so when challenged with *Salmonella typhimurium* or *Toxoplasma gondii*, drastically reducing pathogen translocation and emphasizing the ability of αEβ7 to limit γδTc IEL migration and impact host defense against infection (80). In a study on suckling Lewis rats, probiotics significantly increased both CD62L-positive and negative CD4^−^CD8^−^ T cells expressing αEβ7 in mesenteric lymph nodes; in IEL, significantly increased CD62L^−^ αEβ7-expressing CD4^−^CD8^−^ cells were observed, hypothesized to result from enhanced homing and retention, respectively (81).

## Concluding Remarks

T cells use classical cell biological pathways in new ways (82). Thus, understanding integrin functions on other cell types, including αβTc, suggests but does not dictate their roles on γδTc. Some roles suspected in human γδTc have been confirmed in other species, whereas interspecies variation also exists. Some integrin functions are expected and others surprising, such as HIV-induced Vδ2 demise enabled by α4β7 complexed with CCR5 (60). This review describes the tip of the iceberg with respect to integrins on γδTc; some have yet to be explored at all, and others are worthy of further study. Understanding integrin contributions to γδTc activation, proliferation, and cytotoxicity could inform better expansion protocols and improve γδTc immunotherapy for a variety of indications. We have much to learn about integrin involvement in the myriad functions of these fascinating cells.

## Author Contributions

GS reviewed the literature, wrote the manuscript, and designed both the table and the figure.

## Conflict of Interest Statement

The author declares that the research was conducted in the absence of any commercial or financial relationships that could be construed as a potential conflict of interest.
